# Design and Simulation of a Mass Sensor Using Nanoscale Hf_0.5_Zr_0.5_O_2_ Piezoelectric Membranes with Loading Platform

**DOI:** 10.3390/nano16140862

**Published:** 2026-07-13

**Authors:** Zhicong Li, Haoqi Lyu, Jiahui Xie, Wuhao Yang, Zhuohui Liu, Zhenxiang Qi, Kunfeng Wang, Chen Ge, Xudong Zou

**Affiliations:** 1 State Key Laboratory of Transducer Technology, Aerospace Information Research Institute, Chinese Academy of Sciences, Beijing 100190, China; lizhicong23@mails.ucas.ac.cn (Z.L.); lvhaoqi19@mails.ucas.ac.cn (H.L.); qizhenxiang21@mails.ucas.ac.cn (Z.Q.); wangkunfeng@aircas.ac.cn (K.W.); 2 School of Electronic, Electrical and Communication Engineering, University of Chinese Academy of Sciences, Beijing 100049, China; 3 QiLu Aerospace Information Reasearch Institute, Jinan 250101, China; 4 Beijing National Laboratory for Condensed Matter Physics, Institute of Physics, Chinese Academy of Sciences, Beijing 100190, China; xiejiahui@iphy.ac.cn (J.X.); liuzhuohui@iphy.ac.cn (Z.L.); gechen@iphy.ac.cn (C.G.); 5 School of Electronics and Integrated Circuits, Aerospace Information Technology University, Jinan 250200, China

**Keywords:** NEMS, Hf_0.5_Zr_0.5_O_2_, mass sensor, micro/nano manufacturing, resonator

## Abstract

Resonant mass sensors based on micro/nanoelectromechanical systems (MEMS/NEMS) offer a promising approach for label-free gravimetric detection. However, practical applications often require not only high sensitivity but also improved loading repeatability and reduced dependence on mass loading position. In this work, a suspended resonant mass sensor based on a 10 nm-thick Hf_0.5_Zr_0.5_O_2_ (HZO) piezoelectric film is proposed. A central silicon loading platform is introduced to provide a mechanically robust and spatially uniform sensing region. A Kirchhoff plate model incorporating residual stress is established to analyze the effects of residual stress and platform geometry on the resonant characteristics. The device is fabricated by combining SOI micromachining with wet transfer of the ultrathin HZO film. Laser Doppler vibrometry measurements show a first-order resonant frequency of 1.303 MHz and a quality factor of 342, corresponding to an extracted residual stress of approximately 1.319 GPa. Finite element simulations calibrated by experimental parameters indicate a uniform first-mode displacement distribution and a linear frequency response to added mass from 0 to 1 ng. The obtained mass sensitivities are 150.7 Hz/pg and 166.8 Hz/pg from finite element and analytical models, respectively. The proposed structure provides a feasible route toward repeatable pg-level resonant mass sensing based on ultrathin piezoelectric films.

## 1. Introduction

Resonant mass sensors convert mass loading into measurable resonance frequency shifts and have become an important platform for ultrasensitive detection of particles, thin films, biomolecules, and chemical species. Owing to their small effective modal mass and high operating frequency, nanoelectromechanical-system (NEMS) resonators can achieve extremely high mass responsivity and resolution. Over the past two decades, a variety of nanomechanical resonators, including silicon nanobeams, suspended membranes, carbon nanotubes, graphene resonators, and other two-dimensional material devices, have been explored for mass sensing applications [1,2,3,4,5]. In particular, graphene-based NEMS resonators have demonstrated sub-attogram mass resolution at room temperature, while carbon nanotube resonators have approached the single-molecule detection regime [4,5]. These results highlight the unique advantages of nanomechanical resonators for realizing high-frequency, high-sensitivity, and label-free mass sensing.

Despite their excellent sensing performance, practical implementation of nanomechanical mass sensors remains challenging. Most reported NEMS mass sensors rely on optical interferometry, laser Doppler vibrometry, magnetomotive actuation, or capacitive transduction for resonance excitation and readout [6,7,8,9,10]. Optical methods generally require bulky external instrumentation and precise alignment, whereas capacitive transduction often suffers from small signal levels and stringent gap requirements. Furthermore, many ultrathin membrane resonators exhibit strong spatial dependence of mass responsivity, meaning that identical masses deposited at different locations can produce significantly different frequency shifts. Such position-dependent responses reduce measurement repeatability and limit practical applications involving particle deposition, thin-film loading, and surface adsorption [11,12,13].

Recent advances in ultrathin piezoelectric materials provide a promising route toward electrically driven and electrically readout nanomechanical resonators. Hf_0.5_Zr_0.5_O_2_ (HZO) has recently emerged as a promising ferroelectric material for nanoscale electromechanical systems due to its unique combination of CMOS process compatibility and scalable ferroelectricity at nanometer thicknesses. Unlike conventional piezoelectric materials such as AlN and ZnO, which typically require high-temperature deposition processes and offer limited polarization engineering capability, HZO can be integrated within standard back-end-of-line (BEOL) CMOS process flows, enabling heterogeneous integration with silicon electronics at a low thermal budget [14,15,16]. Moreover, HZO is a lead-free ferroelectric material that satisfies RoHS environmental regulations, providing a sustainable alternative to Pb-based ferroelectrics such as PZT, which are constrained by toxicity concerns and CMOS incompatibility [14]. Beyond mass sensing applications, HZO has been widely explored in ferroelectric memories, field-effect transistors, infrared sensors, and nanoelectromechanical transducers, demonstrating stable ferroelectric polarization and strong electromechanical coupling at nanometer-scale thicknesses [15,17]. These properties make HZO particularly attractive for ultrathin MEMS/NEMS resonant devices, where both scalability and monolithic integration are critical requirements. Compared with AlN-based and ZnO-based piezoelectric MEMS resonators, which rely on non-switchable piezoelectricity and often require high-temperature sputtering or epitaxial growth, HZO enables additional design flexibility through ferroelectric polarization engineering. In contrast, PZT exhibits strong piezoelectric coefficients but suffers from lead-related environmental concerns and limited compatibility with CMOS fabrication processes.

In addition, to address the intrinsic limitation of conventional membrane-based resonant mass sensors, where the frequency response strongly depends on the spatial location of the added mass, a central silicon loading platform is introduced in this work. Unlike designs that aim to maximize point mass sensitivity, the proposed structure intentionally introduces a mechanically robust silicon island at the center of the suspended HZO membrane to define a geometrically constrained and repeatable sensing region. The central loading platform serves multiple purposes. First, it provides mechanical reinforcement to prevent local deformation or structural instability during mass deposition. Second, it modifies the modal displacement distribution of the fundamental resonant mode, leading to a more spatially uniform response within the central region. Third, it significantly reduces the variability in frequency shift induced by random mass placement, thereby improving measurement repeatability and practical robustness in real sensing scenarios such as particle adsorption or thin-film deposition [18,19,20].

In this work, we demonstrate a suspended resonant mass sensing platform based on a 10 nm-thick HZO piezoelectric film. The device is fabricated by integrating wet-transferred HZO thin films with an SOI micromachining process, forming a suspended HZO/Si membrane resonator with a central silicon loading platform. The silicon platform is not intended to maximize the ultimate point mass responsivity; instead, it provides a mechanically robust and geometrically defined loading region for reproducible mass loading measurements. An analytical mass response model and finite element simulations are used to investigate the resonance characteristics, three-dimensional displacement distribution, and spatial uniformity of the local mass response. The results indicate that the proposed HZO/Si suspended resonator can serve as a pg-level mass sensing platform for particulate deposition, thin-film loading, surface adsorption, and microdroplet mass change monitoring.

## 2. Design and Modeling

The resonator in this paper is a three-dimensional annular membrane island structure, consisting of a fixed frame, an HZO piezoelectric thin film, and a central loading platform. During operation, the device is driven by piezoelectric excitation to vibrate in its working mode. When a mass to be measured is placed on the loading platform, it changes the effective mass of the device, thereby causing a shift in the resonant frequency. The mass is detected by measuring the frequency shift.

**Figure 1:**
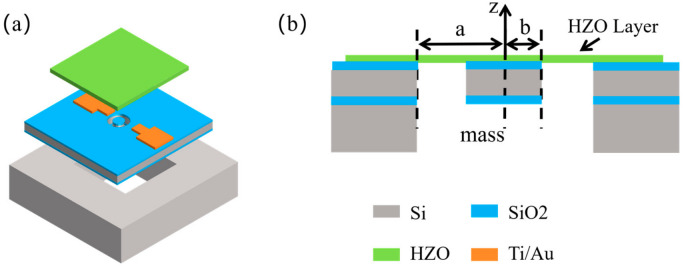
Structural design of mass sensor: (**a**) 3D structure of the mass sensor, (**b**) mass sensor model.

The three-dimensional structure of the device is shown in Figure 1. The device substrate is fabricated using an SOI (Silicon-On-Insulator) wafer, a commonly used substrate material in MEMS devices. The SOI wafer used in this work consists of a 10 μm device layer, a 300 μm handling layer (substrate), and a 0.5 μm thick buried oxide layer in between. The load platform is formed by patterning and etching the top device layer, followed by the transfer of an HZO thin film using a wet transfer method, with the platform and film bonded via van der Waals forces. On the backside, the handling layer (substrate) is etched using a combined dry and wet process to form a cavity larger than the resonator, thereby releasing the resonator.

To simplify the analysis, the classical Kirchhoff thin-plate equation with residual stress is used to derive the influence of residual stress on the resonant frequency. The equation is based on the following fundamental assumptions: (1) the material is linearly elastic and isotropic; (2) the film thickness is constant and much smaller than the in-plane dimensions; (3) the deflection is much smaller than the thickness (small deflection), satisfying linear geometric relationships; (4) the initial residual stress is an in-plane uniform biaxial stress state 14.91(N_0_); (5) the in-plane stretching induced by bending is negligible.

In the equation below, ω is the transverse deflection, D is the bending stiffness of the plate, h is the thickness of, and ν is Poisson’s ratio), $\nabla^{4}$ and $\nabla^{2}$ are the biharmonic operator and the Laplace operator, respectively. Here, N_0_ = σ_0_ is the initial residual stress (membrane force) per unit width.

(1) $$\begin{array}{l} D\nabla^{4}\omega -N_{0}\nabla^{2}\omega =q(r,\theta )\\ \nabla^{4}=\frac{\partial^{2}}{\partial r^{2}}+\frac{1}{r}\frac{\partial }{\partial r}+\frac{1}{r^{2}}\frac{\partial^{2}}{\partial \theta^{2}} \end{array}$$

For a circular diaphragm, the boundary condition is clamped at r = a:

(2) $$\omega (a)=0,{\left.\frac{d\omega }{dr}\right|}_{r=a}=0$$

At r = b, the deflection is ω_0_, and the diaphragm exhibits continuous curvature, yielding

(3) $$\omega \left(b\right)=\omega_{0},{\left.\frac{d\omega }{dr}\right|}_{r=a}=0$$

Furthermore, at r = b, the equilibrium condition gives

(4) $$2\pi \cdot bQ_{r}+F=0$$

where the applied load F and the transverse shear force Q_r_ satisfy

(5) $$\begin{array}{l} F=\left(m+\Delta m\right)g\\ Q_{r}=-D\frac{d}{dr}\nabla^{2}\omega +N_{0}\omega^{\prime } \end{array}$$

where F is the force experienced by the loading platform, and △m denotes the unknown mass to be measured. The solution to the equation is given by

(6) $$\begin{array}{l} \omega (r,N_{0})=\left\{\begin{array}{l} C_{1}-\frac{F}{2\pi \cdot N_{0}}lnr+A_{1}I_{0}(kr)+B_{1}K_{0}(kr),b\leq r\leq a\\ C_{1}-\frac{F}{2\pi \cdot N_{0}}lnb+A_{1}I_{0}(kb)+B_{1}K_{0}(kb),0\leq r\leq b \end{array}\right.\\ k=\frac{N_{0}}{D} \end{array}$$

where I_0_, K_0_ represent the modified Bessel functions of the first kind (since N_0_ > 0). The coefficients A_1_, B_1_, C_1_ can be determined from the boundary conditions.

The resonant frequency can be calculated using the modal function and is given by the following expression:

(7) $$f=\frac{1}{2\pi }\sqrt{\frac{k_{e}}{m_{e}}},\;k_{e}=\frac{F}{\omega_{0}},\;m_{e}=\frac{{\left[\int_{V}\rho \varphi dV\right]}^{2}}{\int_{V}\rho \varphi^{2}dV}$$

where k_e_ and m_e_ are the equivalent stiffness and equivalent mass of the device, respectively, while ω_0_ represents the fundamental frequency. ρ denotes the density distribution function, and ψ is the mode shape function, which can be obtained by normalizing the deflection curve equation.

(8) $$\begin{array}{l} \omega_{0}=\omega (0,N_{0})\\ \varphi =\frac{\omega (r,N_{0})}{\omega_{0}} \end{array}$$

The sensitivity of a mass sensor can be expressed as

(9) $$S_{m}=\frac{\Delta f}{\Delta m}=-\frac{f_{0}}{2m_{eff}}$$

**Figure 2:**
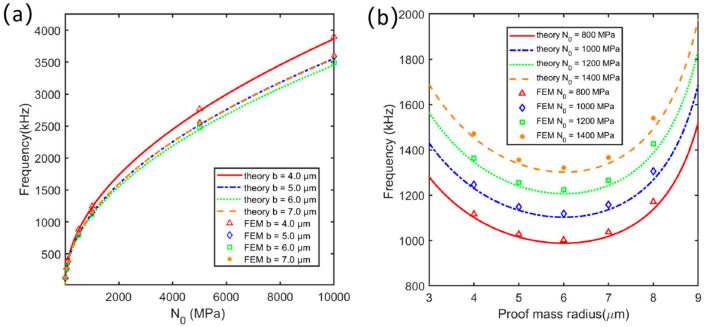
Effects of residual stress and loading platform radius on maximum resonant frequency of mass sensor. (**a**) Isotropic numerical calculation and anisotropic finite element simulation results of effect of residual stress on resonant frequency. (**b**) Isotropic numerical calculation and anisotropic finite element simulation results of effect of loading platform radius on resonant frequency of mass sensor.

The isotropic Kirchhoff plate model is used only for residual stress back-calculation, whereas the orthorhombic phase compliance matrix is introduced later as an approximate material input for estimating the apparent effective piezoelectric coefficient. Therefore, we compared the calculation results of the isotropic model with the anisotropic simulation results. As shown in Figure 2, the error between the model and the finite element simulation is less than 5% within the range of loading platform radius and residual stress that we are concerned with.

According to the relationship between residual stress and resonant frequency shown in Figure 2, the resonant frequency increases with increasing residual stress. This is because the residual stress increases the effective stiffness of the film while having little effect on the mass of the system. Based on the proportional relationship between resonant frequency and the square root of stiffness, the increase in stiffness directly leads to a rise in the first-order modal resonant frequency with increasing residual stress. The resonant frequency exhibits a trend of first decreasing and then increasing with the radius of the loading platform, which is the result of competition between the stiffness enhancement effect and the mass loading effect. When the radius of the cylindrical loading platform is small, increasing the radius mainly adds to the inertial mass of the system, while the effective stiffness of the film changes little; therefore, the first-order resonant frequency decreases with increasing radius. When the radius becomes large enough that the loading platform covers a significant central area of the film, the deformable annular region of the film narrows considerably, causing the effective stiffness to increase sharply. As the rate of this stiffness increase exceeds the effect of mass addition, the frequency turns to rise, exhibiting an overall trend of first decreasing and then increasing.

The radial and transverse bending stresses in the thin film are given by

(10) $$\left\{\begin{array}{l} \sigma_{rr}=-\frac{zE}{1-v^{2}}\left(\frac{d^{2}\omega }{dr^{2}}+\frac{v}{r}\frac{d\omega }{dr}\right)\\ \sigma_{\theta \theta }=-\frac{zE}{1-v^{2}}\left(v\frac{d^{2}\omega }{dr^{2}}+\frac{1}{r}\frac{d\omega }{dr}\right)=v\sigma_{rr} \end{array}\right.$$

The total bending stress (von Mises stress) can be calculated by the following formula:

(11) $$\sigma_{mise}=\sqrt{\sigma_{rr}^{2}+\sigma_{\theta \theta }^{2}-\sigma_{rr}\sigma_{\theta \theta }}$$

The residual stress and the total stress in the thin film are

(12) $$\begin{array}{l} \sigma_{m}=\frac{N_{0}}{h}\\ \sigma =\sigma_{m}+\sigma_{mise} \end{array}$$

Under open-circuit or charge-collection conditions, no voltage is applied to the electrodes, and the electric field E_z_ is very small. Furthermore, in the operating mode of the device, the primary deformation of the film is radial and tangential stretching or compression, rather than direct compression in the thickness direction. Therefore, the piezoelectric coefficient d_31_ plays a dominant role.

(13) $$D_{z}=d_{31}(\sigma_{rr}+\sigma_{\theta \theta })+d_{33}\sigma_{z}+\varepsilon_{33}^{T}E_{Z}\approx d_{31}(\sigma_{\mathrm{rr}}+\sigma_{\theta \theta })$$

On an electrode spanning a 90° circular arc segment, the collected charge is

(14) $$\begin{array}{l} Q=\iint_{A}D_{z}dA=\int_{r_{1}}^{r_{2}}\int_{\theta }d_{31}\left(\sigma_{rr}(r)+\sigma_{\theta \theta }(r)\right)rd\theta dr\\ =d_{31}\frac{\pi }{2}\int_{r_{1}}^{r_{2}}\left(\sigma_{rr}(r)+\sigma_{\theta \theta }(r)\right)rdr\\ =(1+v)d_{31}\frac{\pi }{2}\int_{r_{1}}^{r_{2}}\sigma_{rr}(r)rdr \end{array}$$

In order to simulate the operating characteristics of the device using finite element simulation, it is also necessary to extract the piezoelectric constant d_31_ of the thin film. In this paper, we calculate the apparent effective electromechanical coupling coefficient k_eff,app_ by measuring the electrical transfer response of the device at the resonant frequency, and then estimate the piezoelectric constant. By analyzing the electrical transfer response, we can obtain the resonant frequency f_s_ and anti-resonant frequency f_p_ of the device. The apparent effective electromechanical coupling coefficient is given by [21]

(15) $$k_{eff,app}^{2}=\frac{f_{p}^{2}-f_{s}^{2}}{f_{p}^{2}}$$

It should be noted that the parameter measured in this work is not the rigorous electromechanical coupling coefficient, but rather a mode-dependent and port-dependent apparent effective electromechanical coupling coefficient k_eff,app_. The reason is that the rigorous definition of the electromechanical coupling coefficient essentially corresponds to the energy conversion capability between electrical and mechanical energy, whereas k_eff,app_ extracted from the resonant/anti-resonant frequencies only possesses clear physical meaning under conditions where a single mode dominates and an equivalent one-port model is valid. Furthermore, the anti-resonant frequency is sensitive to parallel capacitance, parasitic capacitance, and external matching networks. Therefore, the k_eff,app_ extracted in this work is used solely to characterize the relative coupling strength of the given mode at the given port, as well as for comparison between different devices or different states. It is neither directly equivalent to the intrinsic piezoelectric coefficient of the material nor used as the sole basis to inversely determine d_31_.

Then, by consulting the compliance coefficient and dielectric constant of HZO, the apparent effective piezoelectric coefficient d_31,app_ can be calculated.

(16) $$k_{ij,app}=\frac{d_{ij,app}}{\sqrt{\varepsilon_{ij}^{T}\cdot s_{ij}^{T}}}$$

## 3. Fabrication

HZO thin films with thicknesses of 10 nm and an La_0.8_Sr_0.2_MnO_3_(LSMO) buffer layer with a thickness of 20 nm were grown by pulsed laser deposition on SrTiO_3_(STO) (001) substrates. An XeCl excimer laser with a wavelength of 308 nm was used, and the imaged laser spot size was 4.0 mm^2^. The LSMO layer and HZO layer were grown under oxygen pressures of 25 and 13 Pa, respectively. A growth temperature of 750°C, a laser fluence of 1.25 J cm^−2^, and a repetition rate of 2 Hz were employed to grow both layers. After deposition, the heterostructure was cooled down to room temperature at a rate of 10 °C min^−1^ under an oxygen pressure of 10 kPa [17].

**Figure 3:**
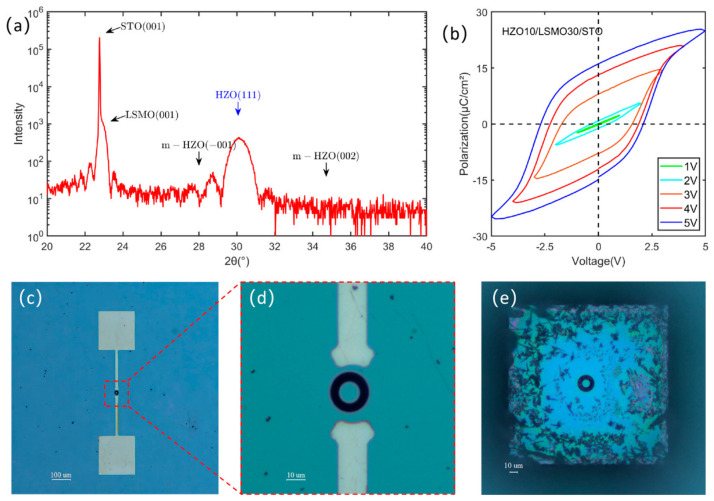
Characterization of HZO thin film and mass sensor. (**a**) X-ray diffraction measurement of the 10 nm HZO film grown on LSMO/STO(La_0.8_Sr_0.2_MnO_3_/SrTiO_3_) substrate. (**b**) P–E hysteresis loops of the 10 nm HZO thin film measured at voltages from 1 V to 5 V. (**c**) Optical microscope image of the device front side (50× *g* magnification). (**d**) Optical microscope image of the device front side (500× *g* magnification). (**e**) Optical microscope image of the device back side (200× *g* magnification).

Prior to transfer, X-ray diffraction (XRD) measurements were conducted using a high-resolution X-ray diffractometer (Rigaku Smartlab, Tokyo, Japan) to characterize the structure and orientation of the film. Specular θ-2θ scans were performed in line-focus mode with an incident beam to determine lattice parameters, using step widths ranging from 0.02° to 0.05°. As shown in Figure 3a, the peak at 2θ = 30° [17] corresponds to the substrate materials LSMO and STO. The dominant Bragg peak near 2θ = 30° is identified as the ferroelectric (111) phase. In addition, the peak for the monoclinic m-HZO (002) phase is nearly undetectable in the pattern. These results indicate that the ferroelectric (111) phase is the predominant phase in the sample used for the device in this study.

The HZO thin film utilized in this device exhibits stable ferroelectricity under repeated mechanical bending (2000 bending cycles) and across a broad temperature range (100K to 400K) [17]. The polarization characteristics of the Pt/HZO/LSMO capacitor were evaluated via P-E hysteresis measurements using a precision ferroelectric analyzer (RADIANT Technologies Inc., Tokyo, Japan), with a 10 kHz triangular pulse applied. The test sample was prepared by depositing a 50 nm Pt dot electrode via magnetron sputtering. A 30 µm diameter photoresist pattern was first defined by standard photolithography. Subsequently, a 50 nm Pt layer was deposited by magnetron sputtering, followed by a lift-off process to form the patterned Pt dot electrodes. As shown in Figure 3b, the hysteresis loops measured at different voltages yield a positive remanent polarization +P_r_ of 16.10 μC/cm^2^ a negative remanent polarization −P_r_ of −14.91 μC/cm^2^, a positive coercive field +E_c_ of 2.09 V, and a negative coercive field −E_c_ of −2.60 V, demonstrating excellent ferroelectric performance.

**Figure 4:**
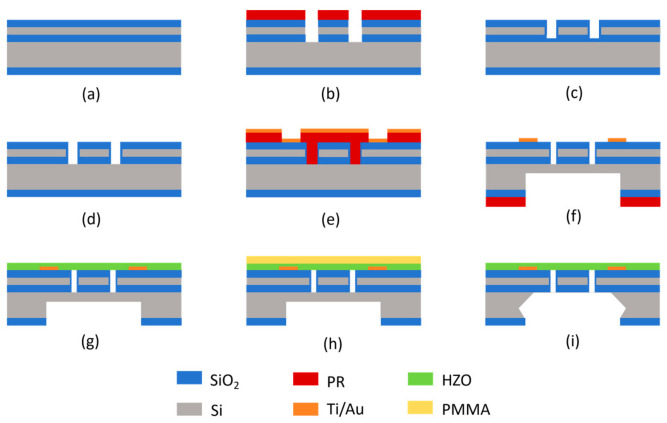
Mass sensor process flow diagram. (**a**) SOI wafer preparation and thermal oxidation. (**b**) Photolithography and RIE/DRIE of loading platform. (**c**) Sidewall thermal oxidation for protection layer. (**d**) RIE of the SiO_2_ on the bottom of trench. (**e**) Ti/Au deposition and lift-off process. (**f**) Backside RIE/DRIE for cavity formation. (**g**) Wafer-dicing and wet transfer of HZO thin films. (**h**) Spin-coat a PMMA protective layer. (**i**) KOH etching.

The HZO film was released from the STO substrate by selectively etching the sacrificial LSMO layer. The freestanding film was then detached by slowly immersing the substrate into deionized water. It is important to note that the sample should be placed horizontally and immersed smoothly into the water. During this process, deionized water gradually penetrates the gap between the HZO film and the substrate, displacing the original etchant. When performed correctly, the released film floats on the water surface. The released film will be transferred onto the device in the step shown in Figure 4g.

During the transfer of the HZO thin film, the target SOI substrate was carefully and slowly inserted into water at a tilted angle and aligned with the predefined device regions. The substrate was then gently lifted out, and the film was transferred onto the substrate via van der Waals adhesion. Subsequently, the transferred sample was naturally dried at room temperature and baked on a hot plate at 60 °C for 30 min to enhance the interfacial adhesion between the HZO film and the silicon-based substrate. These steps ensure the stable integration of the ultrathin HZO membrane during the transfer process without introducing bubbles, mechanical damage, or delamination.

On both the top and bottom surfaces of an SOI wafer with a device layer, buried oxide layer, and handle layer of 10 μm, 500 nm, and 300 μm, respectively, a 1 μm SiO_2_ layer is grown by LPCVD, as shown in Figure 4a. A layer of photoresist is spin-coated on the top surface, and the loading platform region is patterned using photolithography. The surface oxide layer, the SiO_2_ of the buried oxide layer, and the Si of the device layer are then etched by reactive ion etching (RIE) and deep reactive ion etching (DRIE), respectively, as shown in Figure 4b. Subsequently, the fluorocarbon residue left on the sidewalls by DRIE is removed by oxygen plasma cleaning at 300 W for 180 s. A 500 nm SiO_2_ layer is then grown on the sidewalls by thermal oxidation to serve as a protective layer for the subsequent KOH wet etching, as shown in Figure 4c, after which the SiO_2_ at the bottom of the trench is etched by RIE, as shown in Figure 4d. After the loading platform is defined, photoresist is spin-coated on the wafer surface, and the metal electrode regions are patterned by photolithography. A 10 nm/50 nm Ti/Au layer is deposited by magnetron sputtering, followed by lift-off in acetone to obtain the metal electrodes, as shown in Figure 4e. Once the metal electrodes are fabricated, photoresist is spin-coated on the backside of the wafer, and the back cavity region is patterned by photolithography. The Si is then etched to a depth of 250 μm by DRIE. After etching, the wafer is diced into 15 mm × 15 mm chips, as shown in Figure 4f. On the device region of each chip, a 10 nm HZO film is transferred via a wet transfer method. After transfer, the chip is dried on a hotplate at 60 °C for 30 min, followed by rapid thermal annealing at 350 °C for 10 min, as shown in Figure 4g. A layer of PMMA is then spin-coated on the HZO surface as a supporting layer, as shown in Figure 4h. The chip is placed in a single-side etching fixture and immersed in a 20% KOH solution at 60 °C for wet etching. After etching is completed, the KOH solution is gently rinsed off with deionized water, and the chip is dried in an oven at 120 °C. Finally, the PMMA supporting layer is removed by sequential cleaning with acetone, alcohol, and deionized water, completing the device fabrication, as shown in Figure 4i.

Although direct long-term stability data for ultrathin ferroelectric HZO in concentrated KOH are limited, its parent oxides HfO_2_ and ZrO_2_ have been reported to exhibit good chemical stability in alkaline environments [22,23,24]. Moreover, to ensure chemical compatibility with the KOH wet etching process, multiple protection strategies were implemented. A PMMA layer was first spin-coated on the HZO surface to provide temporary physical protection. During wet etching, a single-side etching fixture made of PEEK with PTFE sealing components was used to minimize unintended chemical exposure. Importantly, the HZO film is only exposed to the KOH solution after complete etching of the silicon handle layer, and the total immersion time was strictly controlled to be less than 5 min. These combined measures effectively mitigate potential degradation of the ultrathin HZO film under alkaline conditions.

The optical micrograph of the device is shown in Figure 3d. It can be observed that the device is successfully suspended on the 10 nm HZO thin film. From the backside view in Figure 3e, it is evident that the mass and the substrate have been successfully separated. The green areas in the image correspond to the buried oxide layer, while the crystalline substances visible on the surface are KOH residues precipitated during the drying process. These residues can be removed by rinsing with deionized water.

## 4. Resonance Testing and Finite Element Simulation

**Figure 5:**
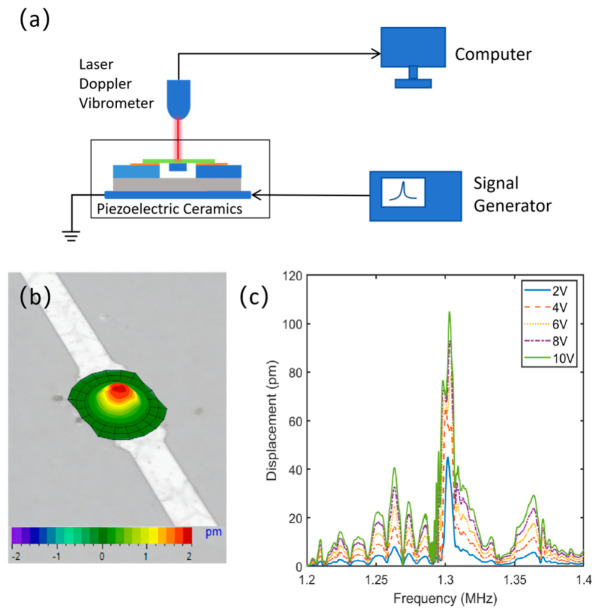
Dynamic performance test of mass sensor: (**a**) test platform; (**b**) vibration mode diagram of mass sensor; (**c**) resonance peak of the first-order mode of mass sensor.

To determine the first-mode resonant frequency of the device and calculate the residual stress within the piezoelectric thin film in the actual device, a laser Doppler vibrometer was first used for testing. The experimental setup is shown in Figure 5a. The device was fixed onto a piezoelectric ceramic, and a signal generator produced a sinusoidal sweep signal to excite the piezoelectric ceramic, which generated periodic mechanical vibrations through the inverse piezoelectric effect, thereby exciting the resonator. The vibrometer used in the experiment (Polytec) integrates a scanning laser Doppler vibrometer, a stroboscopic video microscope, and a scanning white-light interferometer. The laser spot diameter is approximately 2 μm, enabling both single-point and multi-point scanning measurements. The first-mode vibration shape and the amplitude–frequency response of the device obtained from the sweep test are shown in Figure 5b,c respectively. The results indicate that the resonant frequency of the device is 1.303 MHz, and the quality factor Q of the resonant peak is 342. Substituting the measured first-mode resonant frequency into the previously established mathematical model yields a residual stress of 1.319 GPa for the HZO piezoelectric thin film.

**Figure 6:**
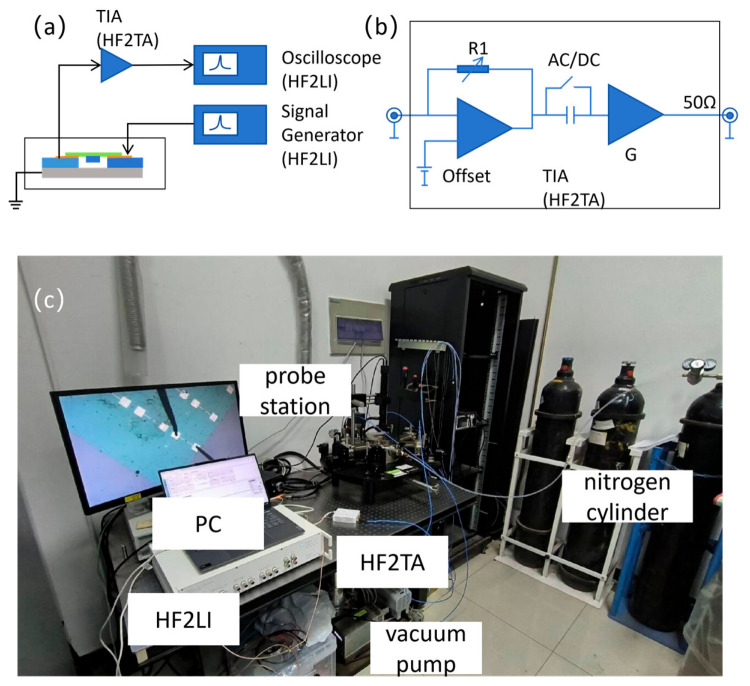
Electrical resonance peak measurement setup. (**a**) Diagram of the electrical resonance peak measurement setup. (**b**) Circuit diagram of HF2TA. (**c**) Electrical resonance peak measurement experimental platform.

To preliminarily verify the feasibility of the device in actual testing and to extract the apparent effective piezoelectric coefficient of the piezoelectric thin film, a voltage sweep test was conducted on the device. The experimental setup is shown in Figure 6a. The voltage sweep signal was provided by a Zurich Instruments lock-in amplifier, model HF2LI. The output signal from the device was amplified by a transimpedance amplifier (HF2TA) and then fed into the HF2LI lock-in amplifier for analysis. The circuit diagram of the transimpedance amplifier is shown in Figure 6b.

**Figure 7:**
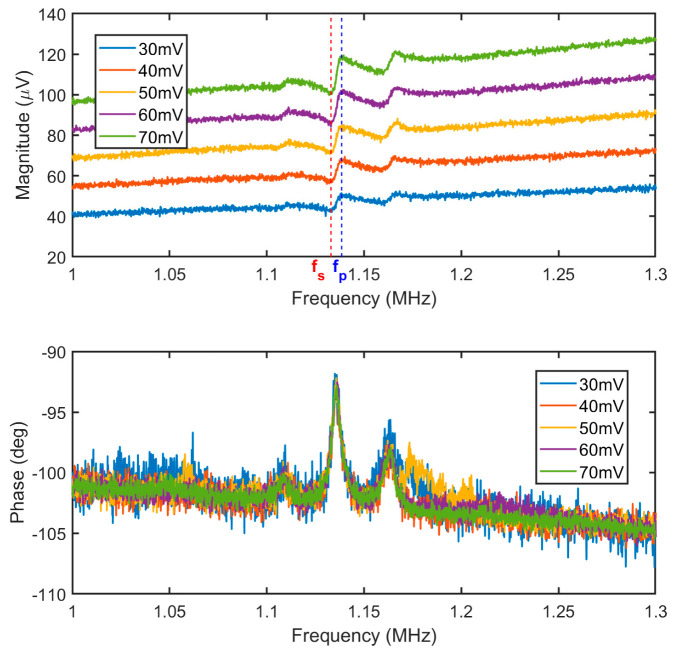
Apparent resonance and anti-resonance features extracted from the electrical transfer response.

A frequency sweep was performed near the first-mode resonant frequency of the device to locate the resonant peak of the first mode under electrical excitation. The measured voltage output signal is shown in Figure 7. Analysis of the experimentally measured resonant peak yielded a series resonant frequency f_s_ of 1.1328 MHz and a parallel resonant frequency f_p_ of 1.1385 MHz. Subsequently, the apparent effective electromechanical coupling coefficient k_eff,app_ of the piezoelectric thin film was calculated using Formula (15). Due to the influence of parasitic capacitance, shunt capacitance, wiring/probe effects, local electric field distribution, or system matching networks, the resonant frequency measured from the electrical transmission response is lower than that measured by the laser vibrometer. Therefore, the extracted coupling coefficient represents a device-level apparent parameter rather than an intrinsic material property. The compliance matrix of the HZO thin film can be obtained by querying the elastic dataset of the Materials Project [25,26]:

(17) $$S=\left(\begin{array}{cccccc} S_{11} & S_{12} & S_{13} & 0 & 0 & 0\\ S_{12} & S_{22} & S_{23} & 0 & 0 & 0\\ S_{13} & S_{23} & S_{33} & 0 & 0 & 0\\ 0 & 0 & 0 & S_{44} & 0 & 0\\ 0 & 0 & 0 & 0 & S_{55} & 0\\ 0 & 0 & 0 & 0 & 0 & S_{66} \end{array}\right)=\left(\begin{array}{cccccc} 3 & 0 & -1 & 0 & 0 & 0\\ 0 & 3 & -1 & 0 & 0 & 0\\ -1 & -1 & 3 & 0 & 0 & 0\\ 0 & 0 & 0 & 12 & 0 & 0\\ 0 & 0 & 0 & 0 & 8 & 0\\ 0 & 0 & 0 & 0 & 0 & 24 \end{array}\right)$$

The relative permittivity of ferroelectric orthorhombic HZO was taken in the range of 30–40 [18]. According to Formula (16), the apparent effective piezoelectric coefficient d_31,app_ is calculated to be −2.82~−3.26 pC/N. The negative sign follows the adopted piezoelectric sign convention for the in-plane 31 mode, whereas the magnitude is estimated from the extracted apparent coupling factor. In the simulation, we set the relative permittivity to 35, and the corresponding apparent effective piezoelectric coefficient d_31,app_ is −3.05 pC/N.

**Figure 8:**
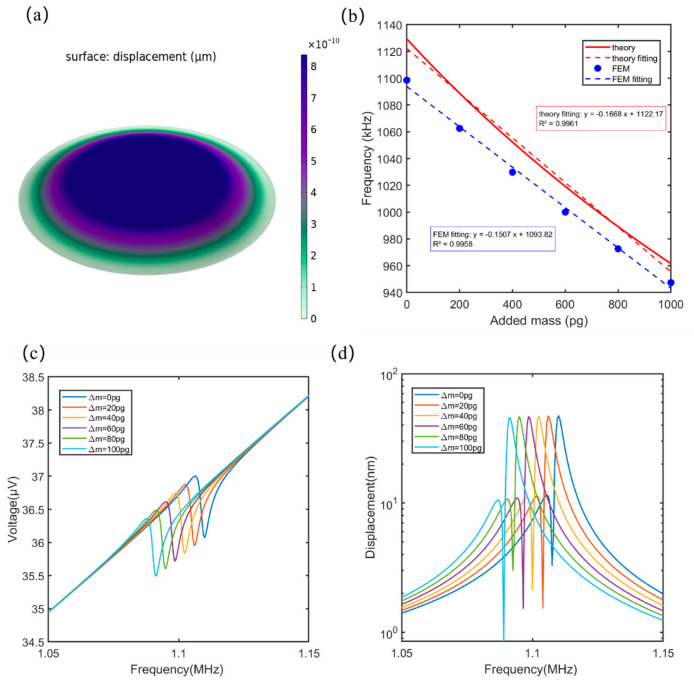
Finite element simulation results of the resonant mass sensor. (**a**) First-mode vibration shape of the device. (**b**) Simulated relationship between added mass and frequency shift. (**c**) Simulated voltage sweep resonant peaks under different added masses. (**d**) Simulated displacement-spectrum resonant peaks under different added masses.

Finally, based on the experimentally extracted parameters, a finite element model was established to simulate and predict the device performance. The simulation results are shown in the Figures. Figure 8a presents the first-mode vibration shape of the device, where it can be observed that due to the constraints of the loading platform, the displacement uniformity in the central region of the film is good. The simulation of the added mass–frequency shift relationship is shown in Figure 8b. Within the range of 0–1 ng, the frequency shift exhibits a good linear relationship with the added mass. The sensitivities obtained from the finite element simulation and numerical calculation are 150.7 Hz/pg and 166.8 Hz/pg, respectively, with the difference mainly attributed to the isotropic approximation of the piezoelectric film’s stiffness matrix in the numerical model. The maximum relative deviations of the response curves from the fitted straight lines are 0.45% and 0.64%, respectively. Figure 8c,d show the finite element simulation results of the voltage sweep resonant peak and the displacement spectrum resonant peak of the device under different added masses, respectively.

## 5. Discussion

The proposed device can be regarded as an intermediate platform between conventional MEMS resonant mass sensors and NEMS mass sensors. Conventional MEMS mass sensors commonly employ piezoelectric thin films such as AlN or ZnO, which provide convenient electrical transduction but usually have micrometer-scale structural dimensions [27,28]. In contrast, NEMS mass sensors based on graphene, carbon nanotubes, or MoS_2_ can achieve extremely small effective mass, but they often face challenges in large-scale fabrication, device-to-device uniformity, and practical electrical readout. The HZO-based resonant sensor proposed in this work attempts to bridge these two regimes by introducing an ultrathin nanometer-scale ferroelectric layer into an SOI-MEMS suspended structure. Therefore, the contribution of this work lies not only in the absolute resonant frequency, quality factor, or mass sensitivity, but also in demonstrating a feasible route for extending MEMS mass sensing toward nanoscale functional transduction while preserving silicon process compatibility and electrical readout capability.

It should be noted that the current fabrication process still involves non-standard laboratory-level steps, mainly including the wet transfer of the ultrathin HZO film and the release of the suspended central loading platform. These steps require careful process control and are not yet fully compatible with standardized commercial SOI-MEMS foundry services. Nevertheless, the main structural definition of the device, including photolithography, DRIE, metallization, and backside cavity formation, is based on conventional SOI-MEMS processes. In future work, the HZO layer may be further developed as a post-process functional thin-film module integrated after the completion of the standard MEMS structure. This provides a possible route toward foundry-compatible HZO-based MEMS/NEMS resonant mass sensors.

## 6. Conclusions

In this work, a suspended resonant mass sensor based on a 10 nm-thick Hf_0.5_Zr_0.5_O_2_ piezoelectric membrane and a central silicon loading platform was designed, fabricated, and evaluated. Different from resonant sensors that mainly pursue ultimate point mass responsivity, the proposed structure uses the loading platform to define a robust and spatially uniform mass loading region, thereby improving the repeatability of mass-induced frequency shift measurements.

A Kirchhoff plate model incorporating residual tensile stress was established to describe the resonance behavior of the membrane platform structure. The model shows that the resonant frequency is strongly affected by residual stress and that the loading platform radius introduces a trade-off between increased inertial mass and enhanced effective stiffness of the annular membrane. This provides a useful design guideline for balancing mass sensitivity, mechanical robustness, and spatial uniformity. The analytical results agree with finite element simulations within 5% over the investigated parameter range.

The device was successfully fabricated by combining SOI micromachining with wet transfer of an ultrathin HZO film. Laser Doppler vibrometry confirmed the first-order resonant mode at 1.303 MHz with a quality factor of 342 under ambient conditions, and the residual stress in the HZO membrane was extracted to be approximately 1.319 GPa. Electrical frequency sweep measurements further verified the electromechanical response of the transferred HZO membrane and yielded an apparent effective piezoelectric coefficient d_31,app_ of approximately −2.82 to −3.26 pC/N.

Based on the experimentally calibrated parameters, finite element simulations show that the first-order mode provides a relatively uniform displacement distribution within the central loading region. The added mass response remains linear from 0 to 1 ng, with mass sensitivities of 150.7 Hz/pg from finite element simulation and 166.8 Hz/pg from the analytical model. The maximum deviation from linear fitting is below 0.64%, indicating that the proposed membrane platform structure can support repeatable pg-level resonant mass sensing.

Overall, this study demonstrates the feasibility of using ultrathin HZO piezoelectric membranes as active transduction layers in suspended resonant mass sensors. The combination of nanoscale piezoelectric films, SOI-based suspended structures, and a defined loading platform provides a promising pathway for compact mass sensors targeting particulate monitoring, thin-film loading, surface adsorption, and microdroplet mass change measurements. Future work will focus on controlled mass loading experiments, optimization of the loading platform geometry, improvement of the quality factor through packaging and process refinement, and systematic evaluation of detection limit, repeatability, and environmental stability.

## Data Availability

The compliance matrix S used in this work is supported by the open website links to https://next-gen.materialsproject.org/ (accessed on 21 March 2026). The data that supports the findings of this study are available from the corresponding author upon reasonable request.
